# Vaccination against pancreas disease in Atlantic salmon, *Salmo salar* L., reduces shedding of salmonid alphavirus

**DOI:** 10.1186/s13567-016-0362-9

**Published:** 2016-08-05

**Authors:** Pål Skjold, Ingunn Sommerset, Petter Frost, Stephane Villoing

**Affiliations:** 1 MSD Animal Health Innovation AS, Thormøhlensgate 55, 5008 Bergen, Norway; 2 MSD Animal Health Norge AS, Thormøhlensgate 55, 5008 Bergen, Norway

## Abstract

Salmon pancreas disease virus, often referred to as salmonid alphavirus (SAV), causes pancreas disease (PD) in European salmonids. SAV transmits horizontally from fish shedding virus into the water and ocean currents are believed to be a main contributor of viral spread between marine farms. Vaccination against PD is previously shown to reduce mortality and severity of clinical PD. In this study, we demonstrate that vaccination against PD significantly reduces viral shedding from infected individuals. The results suggest that PD vaccination can be an important tool to reduce the infection pressure, a known key risk for PD outbreaks at neighbouring farms.

## Introduction, methods and results

**Figure 1:**
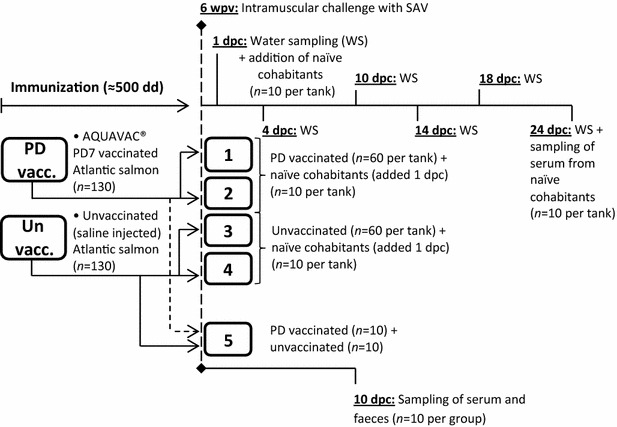
**Illustration of the experimental setup.** 130 Atlantic salmon were vaccinated with AQUAVAC^®^ PD7 into a 500 L tank with flow through freshwater at 12 °C (PD vacc.). As controls, 130 Atlantic salmon were injected with a sterile saline solution and put in a separate tank with same conditions (Un vacc.). Six weeks post vaccination (wpv ≈ 500 degree-days), intramuscular challenge with a Norwegian SAV isolate was performed. Following challenge, 120 fish from each group were allocated in duplicate tanks holding 60 vaccinated or unvaccinated fish each (tanks 1–4). The remaining 10 fish from each group were mixed in a separate tank (tank 5). Water samples (WS) were collected from tanks 1–4 at day 1, 4, 10, 14, 18 and 24 post challenge. 1 day post challenge (dpc), naïve cohabitants were added to tanks 1–4 (*n* = 10 per tank). Serum was sampled from the naïve cohabitants 24 dpc. 10 days post challenge, serum and faeces were sampled from all fish in tank 5 (*n* = 10 per group).

Salmon pancreas disease virus (SPDV), hereafter referred to as salmonid alphavirus (SAV) [1], is a highly contagious virus and the aetiological agent of pancreas disease (PD) in marine reared Atlantic salmon (*Salmo salar*) and rainbow trout (*Oncorhynchus mykiss*) [2, 3], and referred to as sleeping disease (SD) in freshwater reared rainbow trout [4]. Horizontal transmission of SAV has been shown in both fresh- and seawater in experimental trials [5, 6] and experimental challenges have shown that viral shedding typically precedes clinical PD [7, 8]. When clinical PD results in mortality, release of infective virions may also occur from dead fish being subjected to degradation [9]. Current knowledge emphasizes horizontal transmission of SAV by ocean currents in the seawater phase as a main contributor of viral spread within and between farms [10–12]. Hydrodynamic models have been used as a tool to explain the spreading patterns of SAV in fjord systems, and water contact between farms has been pointed out to be the variable that correlates best with field observations of PD-outbreaks in time and space [11, 12]. Vaccination against PD has been shown to reduce the number of outbreaks, cumulative mortality and downgrading at slaughter [13], i.e. reducing the disease severity, although the complete nature of the immune response leading to the observed vaccine efficacy is not known. Both faeces and mucus have been shown to be involved in the shedding of SAV from diseased salmon in experimental trials [8, 14]. Furthermore, analyses of water samples collected during SAV experimental challenge have shown that viral shedding from SAV infected fish coincides with the infection stage when SAV is present in the blood, i.e. during viraemia [7], which occurs prior to histopathological changes in the heart [15]. However, no published studies exist on viral shedding following SAV challenge of PD vaccinated fish. In the current study, we performed a SAV challenge trial where we compared SAV shedding from PD vaccinated versus unvaccinated fish. Presence of SAV was analysed in water samples collected from tanks containing either vaccinated or unvaccinated Atlantic salmon following challenge with a Norwegian SAV isolate. In addition, untreated Atlantic salmon were added to each tank as “detectors” for the presence of infectious SAV in the water. The animal trial design is outlined in Figure 1.

This animal trial was approved by the Norwegian Animal Research Authority (FOTS ID 6100) and carried out at The Industrial and Aquatic Laboratory (ILAB) wet lab facilities at the Bergen High Technology Centre, Norway. The fish population (Atlantic salmon Salmobreed strain) had been pretested for infectious pancreatic necrosis virus (IPNV), piscine orthoreovirus, infectious salmon anaemia virus (ISAV) and SAV by means of real-time RT-PCR (hereafter referred to as qRT-PCR) analysis provided by Patogen Analysis AS (Ålesund, Norway), and found negative. A total of 260 Atlantic salmon parr with an average weight of 30 grams were anaesthetised with FINQUEL^®^ Vet. (ScanAqua AS, Årnes, Norway) according to manufacturer’s recommendations. 130 fish were vaccinated with AQUAVAC^®^ PD7 (0.1 mL/fish), a commercial heptavalent vaccine containing 5 inactivated bacterial antigens (*Aeromonas salmonicida* subsp. *salmonicida*, *Vibrio salmonicida*, *Vibrio anguillarum* serotype O1 and O2a, *Moritella viscosa*) and 2 inactivated viral antigens (IPNV and SAV) in a mineral oil emulsion. As unvaccinated controls, a total of 130 fish were injected intraperitoneally with sterile saline solution (0.9% NaCl, 0.1 mL/fish). The PD vaccinated and unvaccinated groups were kept in individual 500 L tanks with 12 °C flow through fresh water for 6 weeks post vaccination (~500 degree-days) before 120 fish from each group were challenged intramuscularly with 2.05 log_10_ TCID_50_/fish (0.05 mL/fish) of a Norwegian SAV isolate (SAV genotype 3) propagated in Chinook salmon embryo (CHSE-214) cell culture. The fish were, while being challenged, allocated to four 150 L challenge tanks (tank 1 to 4), generating duplicate tanks with 60 fish for both vaccinated and unvaccinated groups. The water flow in the tanks was 300 L per hour.

The remaining 20 fish (10 from the AQUAVAC^®^ PD7 vaccinated group and 10 from the unvaccinated controls) were labelled by maxillae clipping, challenged using the same procedure as above and allocated in one 500 L tank (tank 5) in order to estimate the prevalence of infected individuals in the two groups. These 20 fish were sampled for blood and faeces 10 days post challenge (dpc) which by our experience corresponds to the peak viraemia time point following intramuscular (i.m.) challenge with SAV at 12 °C.

One day post i.m. challenge, 10 untreated, naïve cohabitants were labelled by adipose fin clipping and added to tank 1–4 as “detectors” of infectious SAV shed from the challenged fish already present in the tank. These 40 naïve cohabitants in total were blood sampled at day 24 which, by our experience, corresponds to the peak viraemia time point following cohabitation challenge with SAV at 12 °C.

Water samples from tanks 1–4 were collected 1, 4, 10, 14, 18 and 24 days post challenge using sterile 1 L screw-cap PET bottles (VWR). All samples were vacuum filtered through electropositive Zeta Plus^®^ Virosorb^®^ 1 MDS filters (Cuno Inc., USA) according to a method described in Andersen et al. [7] with minor modifications. Immediately following sampling and prior to filtration each water sample was spiked with 50 μL (5.2 log_10_ TCID_50_) of an ISAV isolate (Bremnes/98) which was used as an exogenous filtration control and for the relative quantification of SAV in the water samples. One litre of filtered and deionized water (Milli-Q^®^, Millipore Corporation), spiked with the same volume of the ISAV suspension, was also subjected to filtration at all sampling points in order to check for potential cross-contamination between water samples. After filtration, individual filters were soaked in 1.4 mL lysis buffer (RNeasy^®^ 96 Kit, Qiagen) for 10 min on a rocker (320 rpm), and 2 × 350 μL (A and B sample) were collected and frozen at −80 °C for subsequent RNA extraction and qRT-PCR analysis. The water samples were analysed by qRT-PCR in triplicate, using the SAV nsP1 [16] and ISAV segment 8 (S8) [17] assays for specific detection of SAV and ISAV, respectively. Only samples that were positive in triplicate were used for normalization performed as described elsewhere [7, 18]. During qRT-PCR setup, positive, negative and no template controls (NTC) were included in all runs. Mean Ct values (Cycle threshold) for the nsP1 assay were normalized against mean Ct values for the ISAV S8 assay using the Microsoft^®^ Excel based software Q-Gene with Eq. 2 [19]. Mean normalized expression (MNE) values were transformed into fold change by calibrating against the lowest MNE value obtained from water samples during the experiment for a better visualization of the data. The cross-contamination controls (pre-filtered and deionized Milli-Q^®^ water) were all negative for SAV and positive for ISAV by qRT-PCR analysis at all sampling points.

Serum (50 μL/fish) and faeces samples (2 × 2 × 2 mm/fish) were spiked with inactivated Equine Influenza Virus (EIV) prior to RNA extraction, serving as an exogenous RNA extraction control and for relative quantification of SAV RNA eventually present in the samples. These samples were analysed by qRT-PCR using the EI H3 assay targeting EIV [20], and the nsP1 assay targeting SAV. Normalised expression (NE) values obtained from serum and faeces were fold changed by calibrating against the lowest NE value obtained for each tissue and log2 transformed for a better visualization of the data. RNA from serum, faeces and water samples was extracted using the RNeasy^®^ 96 Kit (Qiagen) according to the manufacturer’s protocol (elution volume was 50 μL/RNA sample). qRT-PCR analyses were performed using Verso™ 1-step Q-RT-PCR Kit low ROX (Thermo Scientific) using 4 μL of RNA sample. Primer and probe concentrations for each of the three assays were: Fwd. primer: 900 nM, Reverse primer: 900 nM, Probe: 260 nM. Reactions were run in an ABI PRISM^®^ 7500 FAST Thermocycler from Applied Biosystems at the following conditions: 50 °C/30 min, 95 °C/15 min, 40 cycles of 95 °C/15 s and 60 °C/60 s. Standard curve was generated for each assay from dilution series of SAV, ISAV and EIV in Phosphate-buffered saline (PBS). Slope, R^2^ and assay efficiency were calculated for each assay using Q-Gene giving the following values (SAV nsP1/ISAV S8/EI H3): −3.1685/−3.8113/−3.4403 (slope), 0.9964/0.9992/0.9976 (R^2^) and 2.0683/1.8297/1.9529 (E).

Statistical analysis of the effect of PD vaccination on the viral load in the water samples detected by qRT-PCR was performed using the Pair Wise Fixed Reallocation Randomisation Test implemented in the program REST 2009 [21], using as input the mean Ct values of triplicate measurements on each sample for SAV nsP1 assay (target gene), the mean Ct values for the ISAV spike obtained with ISAV S8 assay (reference gene) and the PCR efficiencies of these assays.

Statistical analysis of the effect of PD vaccination on the prevalence of PCR positive fish in serum of the naïve cohabitant fish at 24 dpc was performed using a Generalized Linear Mixed Model (GLMM) for a binomial distribution (SAS procedure GLIMMIX), on the prevalence of positive fish in each tank, including tank as random effect to account for the correlation in the data.

Statistical analysis of the effect of PD vaccination on the viral load detected by qRT-PCR in the serum sampled from the naïve fish was performed using the Pair Wise Fixed Reallocation Randomisation Test implemented in REST 2009, using as input the individual Ct values for SAV nsP1 assay (target gene), the individual Ct values for the EIV spike obtained with EI H3 assay (reference gene) and the PCR efficiencies of these assays. The Ct values used for the analysis in REST 2009 were censored, i.e. the negative samples (with undetermined SAV Ct values) were assigned a Ct = 40 (=max number of cycles per PCR run).

Statistical comparison of the prevalence of SAV positive fish in tank 5 (detected by RT-PCR on serum and faeces samples) between vaccinated and unvaccinated groups was performed by the Fisher’s exact test. Relative Risks were used to determine Relative Percentage Protection [RPP = (1−RR) × 100] as a measure of the vaccine’s efficacy.

For all statistical analysis the level of significance (α) was set at 0.05 and tests were two-sided. Statistical calculations were executed using REST 2009 for the Fixed Reallocation Randomisation Test (set to 2000 iterations), SAS V9.3 for GLMM and SAS Enterprise Guide V7.1 for Fisher test (SAS Institute Inc. Cary NC, USA).

**Figure 2:**
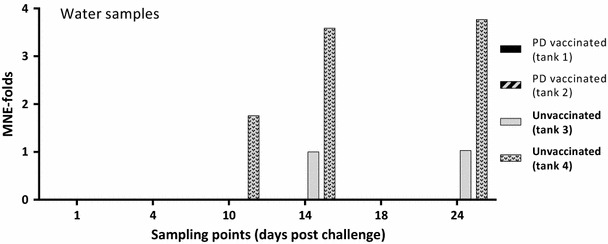
**Relative quantification of SAV in water samples from tanks containing PD vaccinated or unvaccinated fish.** Fold changed mean normalized expression (MNE) values from Ct values obtained from water samples by qRT-PCR. Ct values (mean of triplicate) obtained for the nsP1 assay (target) were normalized against Ct values obtained for the ISAV spike (reference). Subsequently, all MNE values were calibrated against the lowest MNE value obtained from water samples (fold change). Tanks 1 and 2 contained AQUAVAC^®^ PD7 vaccinated fish (*n* = 60 per tank), while tanks 3 and 4 contained unvaccinated fish (*n* = 60 per tank). All four tanks contained naïve cohabitants (*n* = 10 per tank) from 1 dpc.

The ISAV qRT-PCR analysis showed that all water samples were positive for the ISAV spike, demonstrating successful filtration and RNA extraction. SAV mean normalized expression (MNE) values from the water samples (1–24 dpc) are presented in Figure 2. In the water of the two duplicate tanks containing the PD vaccinated and SAV challenged fish (1 and 2) no SAV was detected at any of the six sampling points from 1 to 24 dpc. In the water of the two duplicate tanks containing the unvaccinated and SAV challenged fish (tanks 3 and 4) SAV was detected in the water at 14 and 24 dpc (tank 3) and at 10, 14 and 24 dpc (tank 4). SAV could not be detected in any of the unvaccinated group tanks at 1, 4 and 18 dpc. Statistical analysis of the PCR data obtained for the water samples showed that PD vaccination significantly reduced the SAV viral load in the water (*p* = 0.014).

**Figure 3:**
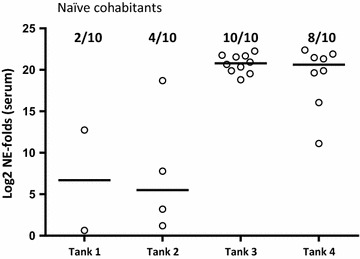
**Relative quantification of SAV in serum of naïve cohabitants 24** **days post challenge.** Log2 of fold changed normalized expression (NE) values from positive, naïve cohabitants (added 1 dpc) for SAV detected by qRT-PCR in serum at 24 dpc (*n* = 10 per tank). SAV nsP1 assay normalized against the exogenous spike EIV assay. NE values are calibrated against the lowest NE value obtained from serum during the experiment and Log2 transformed. Prevalence of positive in each group is shown in fractions. Median values are shown as horizontal lines. In tanks 1 and 2, naïve fish cohabitated AQUAVAC^®^ PD7 vaccinated fish (*n* = 60), while in tanks 3 and 4, naïve fish cohabitated unvaccinated fish (*n* = 60).

Among the 10 naïve cohabitants added to each tank as “detectors” for infectious SAV in the water, low levels of SAV was detected in serum of 2 and 4 fish from tanks 1 and 2, respectively (PD vaccinated fish) while 10 and 8 fish from tanks 3 and 4 (unvaccinated) were positive, respectively (Figure 3). Statistical analysis of the prevalence of PCR positive among the naïve cohabitant fish at 24 dpc showed that there was a significant reduction (*p* = 0.0065) of the prevalence of PCR positives among the naïve fish added to tanks containing PD vaccinated fish compared to the tanks containing unvaccinated fish. Statistical analysis of the SAV Ct values obtained for the naïve cohabitant fish at 24 dpc showed that there was a significant reduction (*p* < 0.0001) of the SAV viral load in the naïve fish added to tanks containing PD vaccinated fish compared to the tanks containing unvaccinated fish.

**Figure 4:**
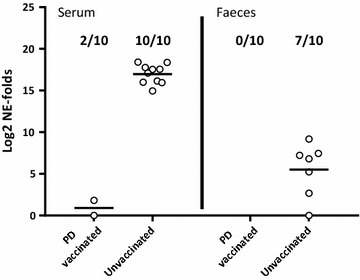
**Relative quantification of SAV in serum and faeces from i.m. challenged PD vaccinated or unvaccinated fish.** Log2 of fold changed normalized expression (NE) values of SAV detected by qRT-PCR in faeces and serum from AQUAVAC^®^ PD7 vaccinated and unvaccinated fish from tank 5. SAV nsP1 assay was normalized against the exogenous spike EIV assay (*n* = 10 per group). NE values are calibrated against the lowest NE value obtained from each tissue during the experiment and Log2 transformed. Prevalence of positive samples in each group is shown in fractions. Median values are shown by horizontal lines. Faeces and serum samples were collected 10 days post i.m. challenge.

In tank 5 containing the mixed challenge control groups, 2 of the 10 PD vaccinated fish were positive for SAV in serum, while none of the same individuals were positive in faeces 10 days after the SAV i.m. challenge (Figure 4). In the unvaccinated fish in the same tank, 10 out of 10 fish were positive for SAV in serum, while 7 out of 10 fish also tested positive for SAV in faeces. Statistical analysis of the PCR data obtained for the fish in tank 5 showed that PD vaccination significantly reduced the viral load in serum and faeces with RPP = 80% (*p* = 0.0007) and RPP = 100% (*p* = 0.0031), respectively.

## Discussion

In this study, we demonstrated that following experimental challenge, PD vaccinated Atlantic salmon shed significantly lower levels of SAV into the water compared to unvaccinated fish. The experimental challenge was done by injection (i.m.) to: (1) avoid possible conflict with waterborne challenge material present in a cohabitation model, (2) have a simultaneous and controlled infection in all test animals, and (3) administer infective virions to muscle/blood stream to avoid possible non-specific protection in the intraperitoneal cavity due to the i.p. vaccination. However, i.m. injection of SAV is probably a severe challenge, as all natural barriers are bypassed and infective virions quickly enter the blood stream to give a rapid systemic infection. SAV challenge by i.m. injection is not the natural route, but its suitability to assess vaccine efficacy has been established [22]. In the water samples collected at six time points between 1 and 24 days post challenge, SAV could, by qRT-PCR, not be detected in either of the two duplicated tanks containing PD vaccinated fish (Figure 2), while all water samples were positive for the ISAV spike confirming valid sample preparation. In the tanks containing unvaccinated fish, SAV was detected in one or both tanks at 10, 14 and 24 days post challenge but not at 1, 4 and 18 dpc. Andersen et al. [7] showed that in water samples collected from tanks containing SAV i.p. injected fish, SAV was detected at day 4, 6, 8, 10 and 13 but not at any later sampling points. As evident from the current study, peak SAV detection in water is somewhat delayed compared to Andersen et al. [7]. In our experience, this is a difference typically seen for i.m. challenge versus i.p. challenge (unpublished). The observed SAV detection kinetics in water is as expected except for the re-occurrence of SAV in tanks 3 and 4 (unvaccinated fish) at day 24. The re-occurrence of SAV in these tanks is probably due to virus shed from the naïve fish added to the tanks as “detectors”. These untreated, naïve fish have been exposed to infective virions shed from the injected fish, corresponding to a classical cohabitant challenge set-up. The time point, 24 days post i.m. challenge of the donor fish and 23 days after adding the naïve fish, corresponds well with expected peak of viraemia (and peak of shedding) following cohabitation challenge at 12 °C [8, 14]. Although, by qRT-PCR, no SAV was detected in any of the water samples from the two tanks containing PD vaccinated fish, some naïve fish added to the tanks were infected (Figure 3). Relatively low concentrations (undetectable by current qRT-PCR method) of infectious SAV must have been present in these two tanks resulting in SAV being detected at low levels in 20 and 40% of the naïve cohabitants at 24 dpc (Figure 3). In comparison, high quantities of SAV (approx. 2^12.5^ fold increase between naïve fish cohabitating PD vaccinated and unvaccinated fish) was detected in 100 and 80% of the naïve fish added to the two tanks containing unvaccinated, i.m. challenged fish. Considering the vaccine efficacy evident in tanks 1 and 2 following i.m. challenge, it is reasonable to presume that if the cohabitants added to these tanks had been PD vaccinated, they would have been protected against this low infection pressure. The results also indicate that a low concentration of infective SAV in the water (undetectable by current qRT-PCR method) is sufficient to infect naïve fish, even when kept under optimal laboratory rearing conditions (low fish density, optimal O_2_/water flow etc.).

In the field, SAV infection pressure and infected neighbouring sites are known to be key risk factors for clinical PD [10, 11]. Infectious SAV has been reported to have a half-life of 1 day at 10 °C in seawater [23], consequently giving the high potential for SAV to spread by passive drift in ocean currents [11, 12]. Adding to this risk is the fact that initial SAV infection happens unnoticed since shedding of SAV from infected animals starts a few weeks after viral exposure [8, 14], while time from infection to clinical disease, the typical indicator for management actions, can be several months [24]. Under field conditions, clinical PD typically emerges in cage after cage within a farm with mortality ongoing up to several months post clinical PD diagnosis [24]. Unlike what is observed in laboratory experiments, individuals within a fish population at a farm will not be infected at the same time. It is therefore likely that a farm population will shed SAV to the water for a longer time period thus adding to the infection pressure within the farm and increasing the probability of transferring SAV to neighbouring sites [10, 11], or beyond by human transport activity.

In conclusion, the present study demonstrates that PD vaccination results in a significant decrease in shedding of SAV to the water if the vaccinated fish become infected. In addition to the PD vaccine efficacy, by means of reduction of disease severity and mortality [13], this vaccine effect can play a major role in reducing the SAV infection pressure within and between farms.
